# Early-Stage Predominance in Breast Cancer Diagnosis Among a Rural Population: Associations With Smoking Status in a 10-Year Registry-Based Cohort Study

**DOI:** 10.7759/cureus.115231

**Published:** 2026-08-26

**Authors:** Matthew T Foley, Allyson V Goeden, Kaylin M Burton, Kyle A Burton, Sheetal Acharya

**Affiliations:** 1 Department of Internal Medicine, Michigan State University College of Human Medicine, East Lansing, USA; 2 Department of Psychology, University of Wisconsin, Madison, USA; 3 Department of Medicine, Michigan State University College of Human Medicine, East Lansing, USA; 4 Department of Oncology, Upper Peninsula Health Systems, Marquette, USA

**Keywords:** breast cancer, cancer screening, health disparities, rural cancer, smoking

## Abstract

Background

Rural populations face unique barriers to preventive care and cancer screening that may influence the timing and stage of breast cancer diagnosis. Although rural residence is frequently associated with delayed cancer presentation, population-based data characterizing diagnostic patterns in rural breast cancer patients, especially in the Upper Peninsula of Michigan, remain limited. This study aimed to explore the distribution of age and stage at breast cancer diagnosis in a predominantly rural population and to examine associations between smoking status, age at diagnosis, and stage of disease presentation.

Methods

We conducted a retrospective, population-based cohort study using the Upper Peninsula Health System (UPHS) tumor registry. The study consisted of women diagnosed with primary breast cancer between January 2013 and December 2023. Variables extracted included age at diagnosis, clinical stage, smoking status, and race. Descriptive statistics characterized demographic and clinical distributions. One-way analysis of variance (ANOVA) with Bonferroni-corrected pairwise comparisons assessed differences in age at diagnosis by smoking status. Chi-square testing evaluated the association between smoking status and stage at diagnosis (early vs. late).

Results

Among 1,061 staged cases, early-stage disease (stage 0, IA, IB) comprised 79.5% of diagnoses, with stage IA alone accounting for 45.3%. Age at diagnosis differed significantly by smoking status (F(3, 1193) = 19.83, p < .001): current smokers were diagnosed a mean of 4.6 years younger than never smokers (58.5 vs. 63.1 years, p < .001) and 8.4 years younger than prior smokers (58.5 vs. 66.9 years, p < .001). Stage at diagnosis also differed by smoking status (χ² = 8.47, p = .037), with current smokers having the highest proportion of late-stage disease (26.2%) compared with never smokers (21.0%) and prior smokers (15.5%). Younger (<40 years) and older (≥80 years) women had the highest rates of late-stage presentation.

Conclusions

In this predominantly rural referral population, breast cancer was diagnosed at early stages in nearly four of five cases. Current smoking was associated with both younger age at diagnosis and a higher proportion of late-stage disease, identifying tobacco use as a potentially modifiable factor influencing breast cancer detection patterns. These findings support continued investment in rural breast cancer screening infrastructure and advocate for integrating tobacco cessation into breast cancer prevention strategies.

## Introduction

Breast cancer remains the most commonly diagnosed cancer among women in the United States, with an estimated 321,910 new cases of invasive breast cancer expected to be diagnosed in 2026 [1]. Advances in mammographic screening and systemic therapy have substantially improved survival over the past three decades, with five-year relative survival exceeding 90% for localized disease [2,3]. However, these gains are not distributed equally across all populations, and significant disparities persist along geographic, socioeconomic, and behavioral lines [4].

Rural populations, in particular, face well-documented barriers to cancer care, including limited availability of screening facilities, shortage of subspecialty providers, greater travel distances, lower health insurance coverage, and higher rates of poverty [4-6]. Multiple studies have reported that rural women are more likely to present with advanced-stage breast cancer and to experience poorer survival outcomes relative to their urban counterparts [5,7]. These findings have reinforced a persistent narrative that rurality is inherently associated with diagnostic delay.

Tobacco use represents a particularly relevant behavioral risk factor in this context. Rural communities in the United States have consistently higher smoking prevalence than urban areas [8], and active smoking likely confers increased breast cancer risk [9]. Despite this evidence, few studies have examined whether smoking status is associated with differences in age at breast cancer diagnosis or stage at presentation within rural populations.

The Upper Peninsula of Michigan is a geographically isolated, predominantly rural region with a population of approximately 300,000, an aging demographic profile, and limited oncologic infrastructure. The majority of the Upper Peninsula's 15 counties are classified as nonmetropolitan under the Office of Management and Budget's metropolitan statistical area definitions, and the region is designated as medically underserved by the Health Resources and Services Administration [10]. Further, the Upper Peninsula has higher-than-state-average smoking rates compared to the state average [11]. Upper Peninsula Health System (UPHS) Marquette serves as a referral center for the region and operates one of only two radiation oncology centers in the Upper Peninsula. The institution maintains accredited breast imaging services and has access to surgery colleagues. State-supported screening assistance programs are also available to eligible residents. Together, these elements create an environment in which system-level capacity may modify anticipated rural disparities. The purpose of this study was twofold: first, to characterize the distribution of age and stage at breast cancer diagnosis in this predominantly rural referral population over a 10-year period; and second, to examine associations between smoking status, age at diagnosis, and stage at presentation.

## Materials and methods

Study design and setting

We conducted a retrospective, registry-based cohort study using data from the UPHS tumor registry in Marquette, Michigan, which included cases first diagnosed or who received the first course of treatment at UPHS Marquette. The study was approved by the Michigan State University Institutional Review Board (STUDY202500913). As the study utilized retrospective, de-identified registry data, informed consent was waived. Individual-level residential address data were not available in the extracted dataset. Therefore, rurality was not directly measured at the patient level. The study population represents patients diagnosed or treated at a regional health system whose catchment area is predominantly rural based on federal designations [10]. The term "predominantly rural population" as used in this manuscript refers to the geographic and demographic characteristics of the region served by UPHS rather than a verified individual-level residential classification. This distinction is acknowledged as a limitation.

Study population

This study used a retrospective, population-based design that captured all eligible cases identified in the UPHS tumor registry over the 10-year study period (January 1, 2013 - December 31, 2023). Because the sample represents a census of the target population rather than a drawn sample, traditional a priori sample size calculations, designed to address sampling error, have limited applicability in this context, and no such calculation was performed prior to data extraction [12]. Inclusion criteria were: (1) female sex; (2) primary breast cancer diagnosis identified by International Classification of Diseases for Oncology, Third Revision (ICD-O-3) topography codes C50.0 through C50.9 [13]; (3) date of diagnosis between January 1, 2013, and December 31, 2023; and (4) first diagnosed or received first course of treatment at UPHS Marquette. There were no exclusions based on age, race, histologic subtype, or treatment received.

Sample size

This study included all eligible cases in the UPHS tumor registry over the 10-year study period rather than a drawn sample. Because the dataset represents a census of the target population, traditional sample size calculations were not applicable, and no sample size formula was used [12]. The final cohort comprised 1,211 cases, of which 1,061 had staging data available from the tumor registry.

Variables

The following variables were extracted from the tumor registry: date of initial diagnosis, primary site (ICD-O-3 topography code [13], age at diagnosis (in years), American Joint Committee on Cancer (AJCC) clinical TNM staging [14,15] (including all T, N, M components and the overall stage group), tobacco use status, and race. Tobacco use was coded as current smoker, prior smoker, never smoker, or unknown, per registry convention. Race was self-reported and categorized according to standard registry codes.

Clinical stage was classified according to the AJCC staging system in effect during each diagnosis year (7th edition for 2013-2017 cases [14]; 8th edition for 2018-2023 cases [15]). For analytic purposes, stages were dichotomized as early (stage 0, IA, IB) or late (stage IIA, IIB, IIIA, IIIB, IIIC, IV). For analytic purposes, stage was dichotomized as early (stage 0, IA, IB) versus late (stage IIA-IV), a threshold based on tumor-size and nodal criteria rather than the stage I-II versus III-IV convention used in some studies. This grouping was defined using the AJCC anatomic stage groups. Under this system, the early category comprises disease with a primary tumor ≤2 cm and, at most, nodal micrometastases, that is, stage 0 (in situ), stage IA (T1N0M0), and stage IB (T0-T1 with N1mi). The late category begins at stage IIA, which is reached upon either macrometastatic axillary nodal involvement (N1: 1-3 nodes with at least one deposit >2.0 mm; e.g., T0-T1N1M0) or a primary tumor >2 cm (T2N0M0) [1]. We selected this cutoff because node-negative, small-tumor disease is most consistent with detection through routine screening mammography, whereas the transition to macrometastatic nodal disease or a larger primary typically prompts more extensive locoregional treatment and reflects a meaningfully different clinical scenario. Clinical stage group assignment for all cases was based on anatomic staging criteria (T, N, M, classification) only. Although the AJCC 8th edition introduced a prognostic staging system incorporating grade, estrogen receptor, progesterone receptor, and HER2 status, this study used only anatomic stage groups, which remained consistent between the 7th and 8th editions for the stage categories used in our dichotomization. The early-versus-late classification (stage 0/IA/IB vs IIA-IV) was applied uniformly across both staging editions without reclassification, and no discontinuity in annual stage distributions was observed at the 2017-2018 transition point. Unstaged cases and cases not eligible for AJCC staging were excluded from the staging analysis but included in overall demographic descriptions.

Statistical analysis

Descriptive statistics were used to characterize the study population, including means, standard deviations, and medians for continuous variables and frequencies and proportions for categorical variables. The primary analysis examined the association between smoking status and age at diagnosis using a one-way analysis of variance (ANOVA). Pairwise comparisons were performed using Bonferroni-corrected t-tests to adjust for multiple comparisons. A secondary analysis examined the association between smoking status and stage at diagnosis (early vs. late) among staged cases with documented smoking status (n = 1,052) using Pearson’s chi-square test. No multivariable adjustment was performed. Given the significant difference in age across smoking groups, age represents an important confounder in the smoking-stage analysis, as age influences both screening behavior and stage at presentation. The limited variability in available covariates (96.6% White race, categorical smoking data without pack-years) precluded meaningful multivariable modeling. The smoking-stage association should then be interpreted as an unadjusted, exploratory finding. Analyses were performed using R (version 4.3; R Foundation for Statistical Computing, Vienna, Austria) and Python (version 3.11; Python Software Foundation, Beaverton, OR, USA).

## Results

Study population characteristics

A total of 1,211 women diagnosed with primary breast cancer were identified during the study period. The mean age at diagnosis was 63.2 years (SD 12.7; median 64; range 18-97). The majority of the cohort was White (96.6%, n = 1,170), consistent with regional demographics. The remaining patients identified as American Indian or Alaska Native (0.8%, n = 10), Asian (0.3%, n = 4), Black (0.1%, n = 1), or unknown (2.1%, n = 26). Regarding tseobacco use, 534 women (44.1%) were never smokers, 297 (24.5%) were prior smokers, 228 (18.8%) were current smokers, and 138 (11.4%) had unknown smoking status. Complete demographic and clinical characteristics are presented in Table 1.

**Table 1:** Demographic and clinical characteristics of the study population. SD, standard deviation.

Characteristic	n	%
Age at diagnosis, years		
Mean (SD)	63.2 (12.7)	—
Median (range)	64 (18–97)	—
Age group		
<40 years	43	3.6
40–49 years	137	11.3
50–59 years	271	22.4
60–69 years	377	31.1
70–79 years	260	21.5
≥80 years	123	10.2
Race		
White	1,170	96.6
American Indian/Alaska Native	10	0.8
Asian	4	0.3
Black	1	0.1
Unknown	26	2.1
Tobacco use status		
Never smoker	534	44.1
Prior smoker	297	24.5
Current smoker	228	18.8
Unknown	138	11.4
Not recorded/blank	14	1.2

Stage at diagnosis

**Figure 1:**
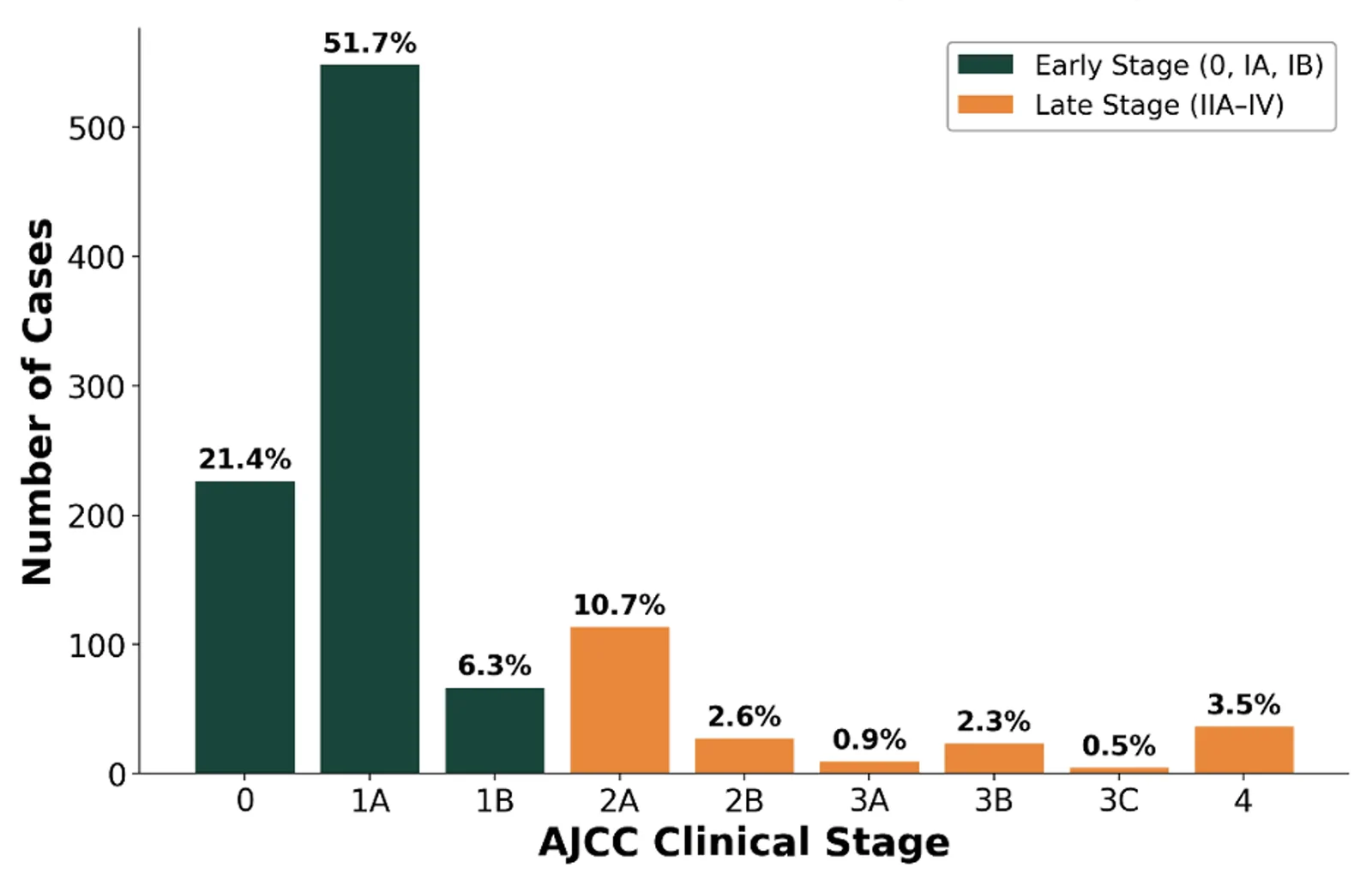
Distribution of clinical stage at breast cancer diagnosis. Bar chart showing the number of cases by American Joint Committee on Cancer (AJCC) clinical stage among 1,061 staged breast cancer cases diagnosed in Michigan’s Upper Peninsula, 2013–2023. Blue bars represent early-stage disease (stage 0, IA, IB); orange bars represent late-stage disease (stage IIA through IV). Percentages are calculated relative to the total cohort (N = 1,211). Early-stage disease comprised 79.5% of staged cases.

Among 1,061 evaluable staged cases (after exclusion of 142 cases coded as not staged, 7 cases not eligible for AJCC staging, and 1 case with a data entry error), early-stage disease (stage 0, IA, or IB) comprised 79.5% (n = 843) of diagnoses, as shown in Figure 1. Stage IA was the single most common presentation, accounting for 45.3% of all cases (n = 549). Stage 0 (in situ) represented 18.7% (n = 227), and stage IB accounted for 5.5% (n = 67). Late-stage disease (stage IIA through IV) was identified in 20.5% (n = 218) of staged cases, with stage IIA being the most common late-stage category (9.4%, n = 114) and stage IV present in 3.1% (n = 37).

Age at diagnosis by smoking status

**Figure 2:**
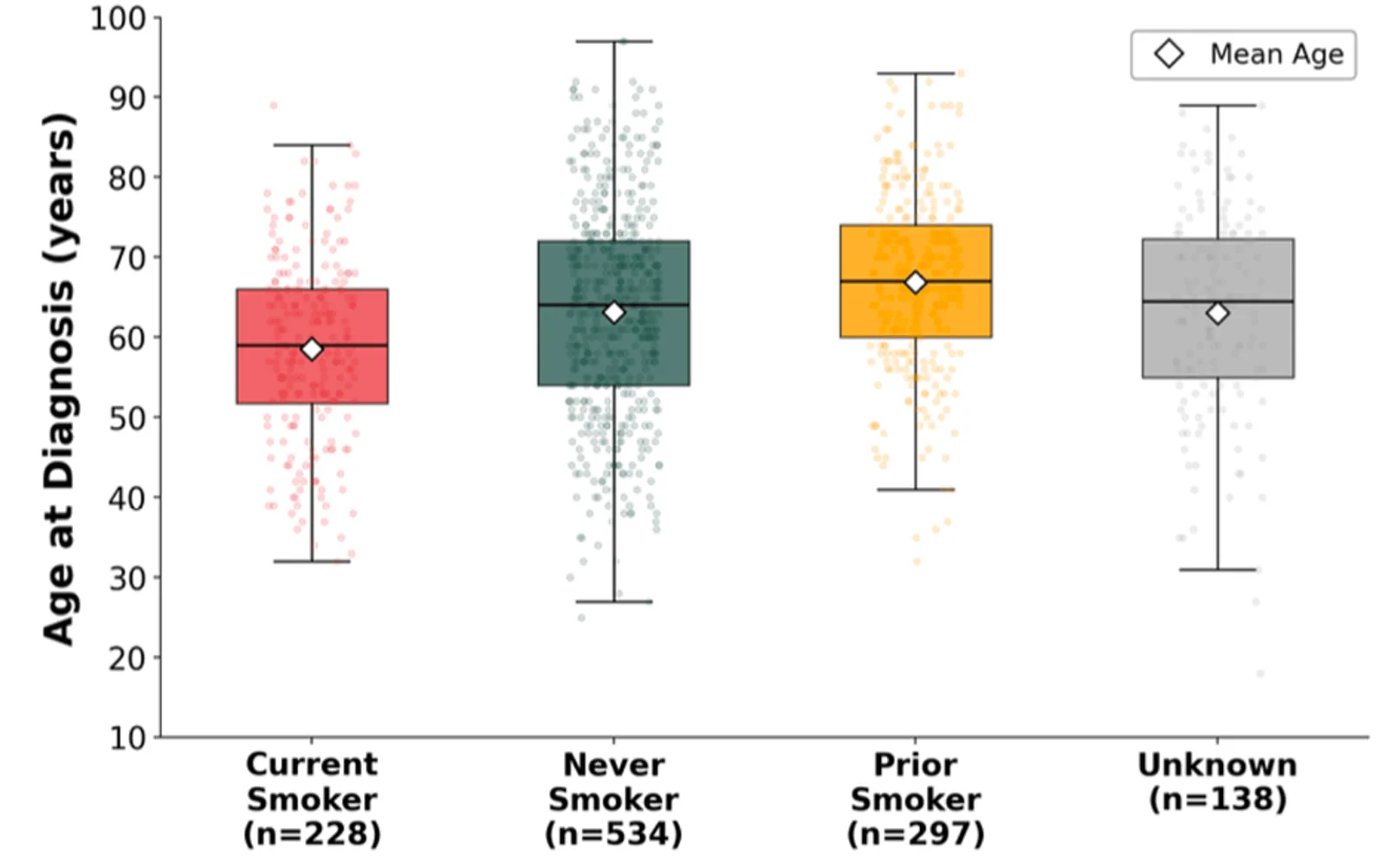
Age at diagnosis by smoking status. Box-and-strip plot showing the distribution of age at breast cancer diagnosis across smoking status categories. Boxes represent interquartile ranges; horizontal lines indicate medians; diamond markers indicate means. Individual observations are displayed as jittered points. Significance brackets indicate Bonferroni-corrected pairwise comparisons. Current smokers were diagnosed at a mean age of 58.5 years, compared with 63.1 years for never smokers and 66.9 years for prior smokers (ANOVA F(3, 1193) = 19.83, p < .001).

Age at diagnosis differed significantly by smoking status (one-way ANOVA: F(3, 1193) = 19.83, p < .001), as shown in Figure 2. Current smokers were diagnosed at the youngest mean age (58.5 years, SD 11.4), followed by unknown (62.7 years, SD 12.9), never smokers (63.1 years, SD 13.3), and prior smokers (66.9 years, SD 11.1). Bonferroni-corrected pairwise comparisons revealed the following significant differences: current smokers were diagnosed 4.6 years earlier than never smokers (p < .001) and 8.4 years earlier than prior smokers (p < .001). Prior smokers were diagnosed 3.8 years older than never smokers (p < .001). No significant difference was observed between never smokers and those with unknown smoking status (mean difference 0.5 years, p = 1.00). Additionally, smoking status was categorized only as never, prior, or current smoker. Data on pack-years or time since smoking cessation were not available and thus could not be incorporated into this analysis.

Stage at diagnosis by smoking status

**Figure 3:**
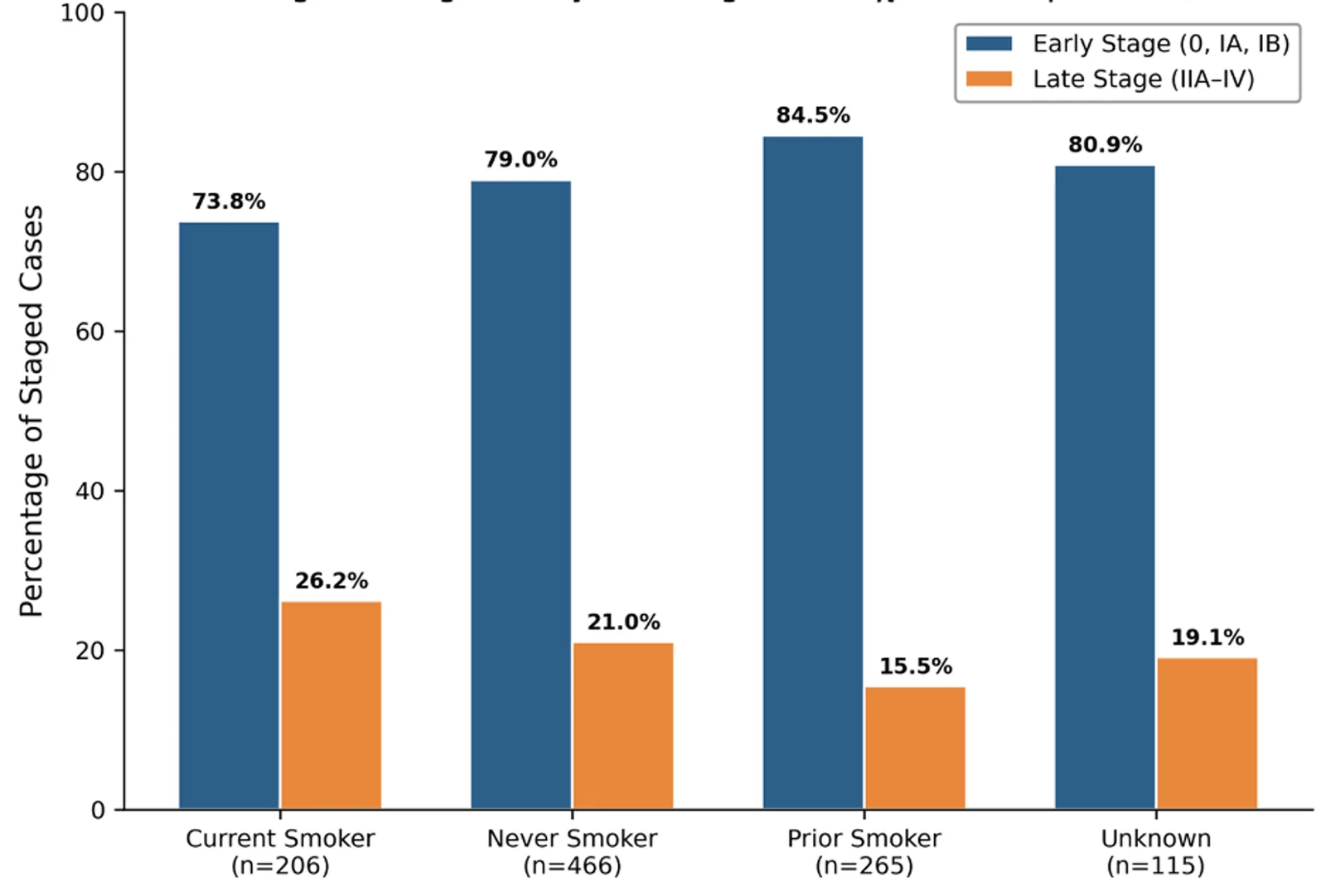
Stage at diagnosis by smoking status. Grouped bar chart comparing the proportion of early-stage (0, IA, IB) and late-stage (IIA–IV) breast cancer by smoking status among 1,052 staged cases with known or unknown smoking status. Current smokers had the highest proportion of late-stage disease (26.2%), while prior smokers had the lowest (15.5%). The association was statistically significant (χ² = 8.47, p = .037).

Among the 1,061 staged cases, 1,052 had documented smoking status (current, never, prior, or unknown) and were included in the chi-square analysis of stage by smoking status. Nine cases with blank/not recorded tobacco status were excluded from this analysis. Smoking status was significantly associated with stage at diagnosis (χ²(3) = 8.47, p = .037), as shown in Figure 3. Current smokers had the highest proportion of late-stage disease at 26.2% (54/206), compared with 21.0% (98/466) for never smokers, 19.1% (22/115) for unknown, and 15.5% (41/265) for prior smokers. The absolute difference in late-stage presentation between current and prior smokers was 10.7 percentage points, representing a clinically meaningful disparity. This analysis, however, was unadjusted. Given the significant age differences across the smoking groups, age may confound the observed smoking-stage association. 

## Discussion

In this study, we observed that the majority of cases (79.5%) were diagnosed at an early clinical stage. This finding is noteworthy given the prevailing literature suggesting that rural populations tend to present with more advanced disease. Additionally, we identified smoking status as a significant factor associated with both younger age at diagnosis and a higher proportion of late-stage disease, findings with direct implications for integrated cancer prevention strategies in rural communities.

The predominance of early-stage diagnosis in our cohort is notable in the context of the broader literature on rural cancer disparities. Prior studies have reported that rural women face higher odds of advanced-stage breast cancer at diagnosis compared with urban women [5]. Further, rural populations have been shown to have higher distance to mammography centers and subsequently increased rates of late-stage breast cancer at diagnosis [16]. The Upper Peninsula Health System has maintained mammographic screening services throughout the study period, and the consistently high proportion of early-stage diagnoses may be related to the availability of screening infrastructure in this region, although this study did not directly measure screening mammography utilization, frequency, or individual screening history. The observed early-stage predominance is consistent with, but does not establish, a role for organized screening. Other unmeasured factors, including referral patterns, tumor biology, and patient self-selection, may also contribute to the observed stage distribution. This interpretation is consistent with research by Anderson et al., who demonstrated that lower breast cancer screening rates in the Appalachia region of the United States were associated with increased later-stage breast cancer diagnosis [17].

In our study, current smokers were diagnosed a mean of 4.6 years younger than never smokers, and this difference was highly significant. Further, our finding that current smokers had the highest proportion of late-stage disease (26.2%) adds a clinically important dimension to the smoking-breast cancer relationship. Prior literature has suggested that tobacco-associated carcinogenesis, mediated through polycyclic aromatic hydrocarbon-induced DNA adducts in breast tissue [18], may accelerate breast cancer development. While this biological plausibility is consistent with the younger age at diagnosis observed among current smokers in our cohort, our retrospective registry-based design cannot establish whether the observed age differences reflect accelerated carcinogenesis, differential screening behavior, or other unmeasured factors. Further, current smokers may be less likely to adhere to recommended screening guidelines as compared to nonsmokers [19]. Both mechanisms could contribute to the observed associations, and this study cannot distinguish between them.

The smoking-stage reported here (p = 0.37) was based on an unadjusted chi-square analysis. Because current smokers were significantly younger than never and prior smokers, and because age independently influences both screening behavior and stage at presentation, the observed association between smoking and late-stage disease may be partly confounded by age. Multivariable analysis adjusting for age, and ideally for screening utilization and socioeconomic factors, would be needed to determine whether smoking status is independently associated with stage at diagnosis in the population.

These results have several practical implications for rural cancer care and prevention. First, the high rate of early-stage detection underscores the value of sustained investment in rural mammographic screening infrastructure. Health systems serving rural populations should view their screening programs as essential components of cancer care, with measurable impact on stage at presentation. Second, the disproportionate burden of late-stage disease among current smokers argues for integrating tobacco cessation into breast cancer screening workflows. Practical models could include coupling cessation counseling or pharmacotherapy referrals with scheduled mammography appointments, an approach that leverages an existing clinical touchpoint to address a modifiable risk factor.

Limitations

Several limitations should be acknowledged. First, this study utilized data from a single regional health system tumor registry, and the results may not be generalizable to other rural populations with different demographic compositions, healthcare access patterns, or screening infrastructure. The study population represents patients diagnosed or treated at UPHS Marquette, a regional referral center serving a predominantly rural catchment area. Individual-level residential rurality was not directly measured, and not all patients in this cohort necessarily reside in rural areas. The designation of this population as "predominantly rural" is based on the geographic characteristics of the region rather than verified patient-level data. Second, the cohort was overwhelmingly white (96.6%), precluding meaningful analysis of racial or ethnic disparities. While this reflects the demographic reality of the Upper Peninsula, it limits the applicability of our findings to more diverse rural populations. Third, smoking status was recorded at the time of cancer diagnosis and does not capture lifetime exposure, pack-years, or timing of cessation. Fourth, the association between smoking status and stage at diagnosis was assessed using an unadjusted chi-square test. Given the significant age difference across smoking groups, age represents an important potential confounder that was not controlled for in this analysis. The observed smoking-stage association should be interpreted as a preliminary finding requiring confirmation through multivariable analysis in future studies with more detailed patient-level data. Additionally, we lacked data on screening mammography utilization, which would have enabled more nuanced analyses. The high proportion of early-stage diagnoses observed in this cohort is consistent with the availability of organized screening, but direct causal attribution to screening programs cannot be established without individual-level screening data. Finally, this is a descriptive study and cannot establish causal relationships between smoking and diagnostic outcomes.

## Conclusions

In this 10-year population-based study of breast cancer in Michigan’s predominantly rural Upper Peninsula, nearly four in five staged cases were diagnosed at an early clinical stage, a finding that may be related to the availability of organized screening infrastructure in this region, although direct causal attribution cannot be established without individual-level screening utilization data. Current smoking status was significantly associated with both younger age at diagnosis and a higher proportion of late-stage disease in unadjusted analyses. These findings support continued investment in rural mammographic screening infrastructure and advocate for the integration of tobacco cessation interventions into breast cancer prevention strategies. Future studies could evaluate longitudinal outcomes, incorporate screening utilization data, include multivariable adjustment for potential confounders, including age, and assess whether integrated smoking cessation-mammography programs improve breast cancer outcomes in rural populations.
